# On-Line Visual Tracking with Occlusion Handling †

**DOI:** 10.3390/s20030929

**Published:** 2020-02-10

**Authors:** Tharindu Rathnayake, Amirali Khodadadian Gostar, Reza Hoseinnezhad, Ruwan Tennakoon, Alireza Bab-Hadiashar

**Affiliations:** 1 School of Engineering, RMIT University, Melbourne VIC 3000, Australia; amirali.khodadadian@rmit.edu.au (A.K.G.); rezah@rmit.edu.au (R.H.); alireza.bab-hadiashar@rmit.edu.au (A.B.-H.); 2 School of Science, RMIT University, Melbourne VIC 3000, Australia; ruwan.tennakoon@rmit.edu.au

**Keywords:** multi-target tracking, random finite sets, GLMB filter, visual tracking, occlusion recovery, occlusion handling

## Abstract

One of the core challenges in visual multi-target tracking is occlusion. This is especially important in applications such as video surveillance and sports analytics. While offline batch processing algorithms can utilise future measurements to handle occlusion effectively, online algorithms have to rely on current and past measurements only. As such, it is markedly more challenging to handle occlusion in online applications. To address this problem, we propagate information over time in a way that it generates a sense of déjà vu when similar visual and motion features are observed. To achieve this, we extend the Generalized Labeled Multi-Bernoulli (GLMB) filter, originally designed for tracking point-sized targets, to be used in visual multi-target tracking. The proposed algorithm includes a novel false alarm detection/removal and label recovery methods capable of reliably recovering tracks that are even lost for a substantial period of time. We compare the performance of the proposed method with the state-of-the-art methods in challenging datasets using standard visual tracking metrics. Our comparisons show that the proposed method performs favourably compared to the state-of-the-art methods, particularly in terms of ID switches and fragmentation metrics which signifies occlusion.

## 1. Introduction

Online visual multi-object tracking is one of the most ubiquitously addressed problems in machine vision with a variety of applications [1,2,3,4,5,6,7,8]. It can be very challenging in its nature. The challenges include estimation of an unknown and time-varying number of objects, continuous state estimation of all objects, discrete combinatorial nature of measurements to objects data association and resolving long and short term occlusions.

A common approach to develop multi-object visual tracking solutions is tracking by detection. In this approach, the outputs of an object detection module are used, usually in conjunction with a data association method, to acquire trajectories of the objects. Two common methods for trajectory extraction are online and batch methods.

Online techniques use the detections in the current and previous frames to estimate the state of the objects at each time epoch [9,10,11,12,13]. In case of miss-detections, they may rely on predictive models to continue tracking until a matching detection is found [14]. The batch methods utilise the extracted information in the entire sequence of frames and iteratively optimise the detection assignment of the current frame using past and future information [15,16,17,18,19,20,21]. Utilisation of both past and future information allows batch methods to manage miss-detections better than online methods [22]. However, such methods cannot be effectively used in applications where multiple objects need to be tracked in real-time. Moreover, other approaches include the algorithm formulated by Pham et al. which relies on multi-camera systems to recover the lost targets [23] and algorithms presented in [8,24] which rely on training a feature representation offline to recover target IDs at the run time.

In order to increase the accuracy of online methods, a proposed solution is to model the objects’ motion as a Markovian process and estimate the object states recursively using a Bayesian filter [1,25,26,27]. In a Bayesian filtering context, tracking by detection is achieved by associating the detections in consecutive frames using temporal information to estimate object trajectories. The central focus is on estimating the number of objects and assigning each object with a unique identity and maintaining it throughout the life of the object.

Many Bayesian filtering algorithms have been developed to tackle the visual multi-object tracking problem, such as particle filter [10,28], joint probabilistic data association filter (JPDAF) [29,30], Marcov chain Mote Carlo (MCMC) data association [31,32], track linking [33,34,35], multiple hypothesis tracking (MHT) [36], kernel based Bayesian filter [37], and Bayesian filters with Relative Motion Network (RMN) [38].

A recent approach to multi-target tracking that has attracted substantial interest is the random finite set (RFS) framework [39]. Motivated by a fundamental consideration in estimation theory–estimation error–this approach represents the collection of target states, called the multi-target state, as a finite set. RFS multi-target filtering techniques such as Gaussian mixture and particle probability hypothesis density filters [26,40,41] have been applied to tracking from video data via detection in [42,43,44]. The more recent RFS based tracking algorithms such as multi-Bernoulli filter [39,45], labeled multi-Bernoulli (LMB) filter [46] and the Generalized labeled multi-Bernoulli (GLMB) filter [47,48] have been applied extensively in multi-object tracking with promising results [25,27,49,50,51,52,53].

Due to the proven accuracy of the GLMB filter (based on the conjugacy of GLMB density priors with standard multi-object likelihood model), various implementations have been proposed in the stochastic signal processing literature to extend the utility of the filter. The LMB filter is an example of such approximations which was applied for tracking to aid intelligent navigation in autonomous cars [54]. Other examples include implementations and approximations developed for multi-target tracking with merged measurements [52], and for extended targets [55], using a particular implementation called δ-GLMB for fusion of RGB-D data for multi-object tracking [56].

In our previous paper [57], we presented a novel filtering solution that was designed based on the GLMB filter but particularly tailored for online visual tracking of multiple targets that can occlude each other. In visual multi-object tracking applications, objects have finite sizes and are commonly represented by rectangular blobs. As such, we proposed an intuitive solution for incorporation of occlusions into stochastic multi-object filters in general, and the GLMB filter in particular.

In order to handle long term occlusion events, we introduced a novel track management scheme which is henceforth referred to as label recovery procedure. In formulating the label recovery procedure, aspects such as the number of time steps between the disappearance and the re-detection of the object, the features of the disappeared and the re-detected object and the spatial distance between the disappeared and the re-detected object were considered. It should be noted that Reuter et al. [58] have proposed a Finite Set Statistics (FISST)-based Multi-target Bayes filter (MTB) which leverage a state dependent multi-target likelihood which account for occlusions. However, unlike the GLMB filter, the MTB filter does not propagate target labels in time and thus cannot handle the problem of label recovery.

Different to our previous paper, this paper provides extensive details of the problem at hand and elaborates on the proposed solution with expansive pseudocodes. Similar to our previous work, we validate our tracking method using publicly available datasets such as PETS [59] and ETH [60] and compare our method against state-of-the-art methods. The comparative results show that our method generally performs better than state-of-the-art methods in terms of common metrics used in visual tracking literature. In this paper, we additionally provide a computational cost analysis and compare the efficiency of the proposed solution to that of the state-of-the-art methods. We also present a comprehensive ablation study of the proposed solution using widely used metrics in computer vision.

The rest of the paper is organised as follows. Section 2 briefly reviews the foundations of RFS filters with a focus on the GLMB filter. Section 3 formulates the occlusion-handling problem in a multi-object filtering context, detailing what the standard filters lack to resolve the issue. We then present our proposed multi-target visual tracking method with occlusion recovery embedded within it, in Section 4. Section 5 presents the comparative results of evaluation of the proposed method and state-of-the-art visual tracking methods on publicly available datasets, followed by concluding remarks presented in Section 6.

## 2. Background

In multi-object tracking, the two main challenges are to estimate the time varying number of objects and to estimate their states. On the other hand, intuitively, a labeled RFS is a set with random cardinality and the elements of that set can also take random values. Moreover, each of the elements in the set is associated with a unique label. We can use the cardinality of an RFS to represent the number of targets, the values of the elements of the RFS to represent the target states, and labels associate with each element to represent the ID of the target. As such, a given multi-object tracking scenario can be comprehensively represented using an RFS. A mathematical formulation of this representation is given below.

Following definitions and notations will be used throughout the rest of this paper. A single-object state is denoted by lower-case letters (e.g., xandx), multi-object states are denoted by upper-case letters (e.g., XandX), spaces are shown by blackboard bold letters (e.g., N,XandL) and labeled entities are denoted by bold letters (e.g., xandX). Furthermore, the standard inner product notation is used as 〈f,g〉≜∫f(x)g(x)dx. The multi-object exponential for a real valued function h(·) raised to a set *X* is defined as $h^{X}≜\prod_{x\in X}h\left(x\right)$, where $h^{\emptyset }≜1$ and the elements of *X* may be of any type such as scalar, vector or set, provided that the function *h* takes an argument of that type. The generalised Kronecker delta function $\delta_{Y}\left(X\right)$ and a generalisation of the inclusion function $1_{Y}\left(X\right)$ are defined in [54] (Table I. Notation). Table 1 lists the other notations used in this paper.

### 2.1. Labeled RFS

A labeled RFS with state space X and discrete label space L is an RFS X on X×L such that L:X×L→L is the projection L((x,ℓ))=ℓ. The finite subset **X** of X×L has distinct labels if and only if **X** and its labels L(X)={L(x):x∈X} have the same cardinality, which can be mathematically denoted as:

$$▵\left(X\right)≜\delta_{\left|X\right|}\left(|L\left(x\right)|\right)=1.$$

The function Δ(X) is called distinct label indicator. The density of a labeled RFS X is a function $\pi :F\left(X\times L\right)\to R^{+}\cup \left\{0\right\}$ that satisfies unit integration over the labeled multi-object state space, i.e.:

$$\int_{X\times L}\pi \left(X\right)\delta X=1$$

In order to append a unique label to each object, each state x∈X is coupled with a unique label $(\ell_{t},\ell_{b})\in L$, where $\ell_{t}$ is the time stamp at which the object has appeared and $\ell_{b}$ is an index to distinguish the objects born at the same time step.

### 2.2. The GLMB Filter

The GLMB filter is formulated based on propagating a particular class of labeled set densities, called GLMB density, through the prediction and update steps of a general Bayesian filtering scheme. A GLMB density is defined on X×L according to [47]:

(1) $$\pi \left(X\right)=\Delta \left(X\right)\sum_{c\in C}w^{\left(c\right)}\left(L\left(X\right)\right){\left[p^{\left(c\right)}\right]}^{X},$$

where C is a discrete index space. The weights $w^{\left(c\right)}\left(L\right)$ and the spatial distributions $p^{\left(c\right)}$ satisfy the normalisation conditions

$$\sum_{L\subseteq L}\sum_{c\in C}w^{\left(c\right)}\left(L\right)=1,\;\int p^{\left(c\right)}\left(x,\ell \right)dx=1.$$

GLMB densities form a class of tractable models for Bayesian inference [61]. Under the standard multi-object model, the GLMB density is closed under the Chapman-Kolmogorov equation and a conjugate prior with standard multi-object likelihood. To implement the GLMB filter in multi-object tracking applications, a particular form of GLMB densities called δ-GLMB density is commonly used as it is explicitly related to track to measurement associations. It is formulated as

(2) $$\pi \left(X\right)=\Delta \left(X\right)\sum_{(I,\xi )\in F(L)\times \Xi }w^{\left(I,\xi \right)}\delta_{I}\left(L\left(X\right)\right){\left[p^{\left(\xi \right)}\right]}^{X},$$

where Ξ is a discrete space representing the history of track to measurement associations and *I* denotes a set of track labels. This distribution can be interpreted as a weighted mixture of exponentials of multi-object densities.

Vo et al. [48] have shown that through the prediction and update steps, a δ-GLMB density maintains its mathematical form as given in Equation (2). Indeed, the prediction step leads to the following δ-GLMB density [48]:

(3) $$\pi \left(X\right)=\Delta \left(X\right)\sum_{\left(I,\xi \right)\in F\left(L_{+}\right)\times \Xi }w_{+}^{\left(I,\xi \right)}\delta_{I}\left(L\left(X\right)\right){\left[p_{+}^{\left(\xi \right)}\right]}^{X},$$

where

(4) $$\begin{array}{cc} w_{+}^{\left(I,\xi \right)} & =w_{S}^{\left(\xi \right)}\left(I\cap L\right)\;w_{B}\left(I\cap B\right), \end{array}$$

(5) $$\begin{array}{cc} p_{+}^{\left(\xi \right)}\left(x,\ell \right) & =1_{L}\left(\ell \right)p_{S}^{\left(\xi \right)}\left(x,\ell \right)+1_{B}\left(\ell \right)p_{B}\left(x,\ell \right), \end{array}$$

(6) $$\begin{array}{cc} w_{S}^{\left(\epsilon \right)}\left(L\right) & ={\left[\eta_{S}^{\left(\xi \right)}\right]}^{L}\sum_{I⊇L}{\left[1-\eta_{S}^{\left(\xi \right)}\right]}^{I-L}w^{\left(I,\xi \right)}, \end{array}$$

(7) $$\begin{array}{cc} \eta_{S}^{\left(\xi \right)}\left(\ell \right) & =\langle p_{S}\left(\cdot ,\ell \right),p^{\left(\xi \right)}\left(\cdot ,\ell \right)\rangle , \end{array}$$

(8) $$\begin{array}{cc} p_{S}^{\left(\xi \right)}\left(x,\ell \right) & =\frac{\langle p_{S}\left(\cdot ,\ell \right)f\left(x|\cdot ,\ell \right),p^{\left(\xi \right)}\left(\cdot ,\ell \right)\rangle }{\eta_{S}^{\left(\xi \right)}\left(\ell \right)}, \end{array}$$

and $p_{S}\left(x,\ell \right)$ denotes the probability of survival for a labeled object with state x=(x,ℓ), f(x|·,ℓ) denotes the single-object state transition density, and $w_{B}\left(I\right)$ and $p_{B}\left(x,\ell \right)$ are the parameters of the following labeled birth density defined as a special case of GLMB density on the birth space B as follows [48]:

(9) $$\pi_{B}\left(X\right)=\Delta \left(X\right)w_{B}\left(X\right){\left[p_{B}\right]}^{X}.$$

The label space is also extended to include the newly born objects, $L_{+}=L\cup B$.

With the standard multi-object likelihood, applying the Bayes’ rule in the update step leads to the following posterior multi-object density that is also of the δ-GLMB mathematical form given in Equation (2) over the extended label space $L_{+}=L\cup B$ that now includes the possible new born targets:

(10) $$\begin{array}{c} \pi \left(X|Z\right)=\Delta \left(X\right)\sum_{I\in F(L_{+})}\sum_{\xi \in \Xi }\sum_{\theta \in \Theta (I)}w^{\left(I,\xi ,\theta \right)}\left(Z\right)\delta_{I}\left(L\left(X\right)\right){\left[p^{\left(\xi ,\theta \right)}\left(\cdot |Z\right)\right]}^{X}, \end{array}$$

where Θ(I) is the subset of current association maps from the label set *I* to the measurement set *Z* in the sense that the object labeled ℓ∈I is associated with measurement $z_{\theta (\ell )}$. For the objects that are missed, by convention θ(ℓ)=0. According to [48] the weights and densities of the updated components of the GLMB density are given by:

(11) $$\begin{array}{cc} w^{\left(I,\xi ,\theta \right)}\left(Z\right) & \propto w_{+}^{\left(I,\xi \right)}{\left[\eta_{Z}^{\left(\xi ,\theta \right)}\right]}^{I}, \end{array}$$

(12) $$\begin{array}{cc} p^{\left(\xi ,\theta \right)}\left(\cdot |Z\right) & =\frac{p_{+}^{\left(\xi \right)}\left(x,\ell \right)\psi_{Z}\left(x,\ell ;\theta \right)}{\eta_{Z}^{\left(\xi ,\theta \right)}\left(\ell \right)}, \end{array}$$

where:

(13) $$\begin{array}{cc} \eta_{Z}^{\left(\xi ,\theta \right)}\left(\ell \right) & =\langle p_{+}^{\left(\xi \right)}\left(\cdot ,\ell \right),\psi_{Z}\left(\cdot ,\ell ;\theta \right)\rangle , \end{array}$$

(14) $$\begin{array}{cc} \psi_{Z}\left(x,\ell ;\theta \right) & =\left\{\begin{array}{cc} 1-p_{D}\left(x,\ell \right)\; & \mathrm{if}\;\;\theta (\ell )=0\\ \frac{p_{D}\left(x,\ell \right)g\left(z_{\theta (\ell )}|x,\ell \right)}{\kappa (z_{\theta (\ell )})}\; & \mathrm{otherwise}, \end{array}\right. \end{array}$$

where g(z|x,ℓ), $p_{D}\left(x,\ell \right)$ and κ(z) are the single-object likelihood, detection probability and clutter intensity function, respectively.

**Remark** **1.** *To avoid exponential explosion of the number of hypotheses, those with very small weights are pruned, and the weights of the remaining hypotheses are renormalised. In our experiments, we set this threshold at $10^{-5}$. Furthermore, if the number of hypotheses after pruning is more than a user defined maximum, we only retain those with the highest weights. In our experiments, we only retained the top 700 hypotheses. In a sequential Monte Carlo (SMC) implementation, the particles representing each single object density $p^{\left(\xi ,\theta \right)}\left(\cdot \right)$ may also need to be resampled. Vo et al. [48] have also suggested a combination of ranked assignment and shortest-path strategies for computationally efficient implementation of the prediction and update steps of the filter.*

**Remark** **2.** *Having a posterior GLMB density in the form of (10), the discrete distribution of number of targets (cardinality) is given by: [48]*

(15) $$\rho \left(n\right)=\sum_{\begin{array}{c} I\in F(L_{+})\\ |I|=n \end{array}}\sum_{\xi \in \Xi }\sum_{\theta \in \Theta (I)}w^{\left(I,\xi ,\theta \right)}\left(Z\right),$$

*and a maximum a posteriori (MAP) estimate for the number of targets is:*

(16) $$\hat{n}=\underset{n}{\arg\max}\;\rho \left(n\right).$$

*Defining:*

(17) $$\left(I^{*},\xi^{*},\theta^{*}\right)=\underset{\begin{array}{c} I\in F\left(L_{+}\right),\;\left|I\right|=\hat{n}\\ \xi \in \Xi ,\;\theta \in \Theta (I) \end{array}}{\arg\max}w^{\left(I,\xi ,\theta \right)}\left(Z\right),$$

*the estimates for object states and their labels are obtained as follows:*

(18) $$\hat{X}={\left\{(\hat{x}\left(\ell \right),\ell )\right\}}_{\ell \in I^{*}},$$

where $\hat{x}\left(\ell \right)=\int x\;p^{\left(\xi^{*},\theta^{*}\right)}\left(x,\ell |Z\right)\;dx.$

## 3. Problem Statement

**Figure 1:**
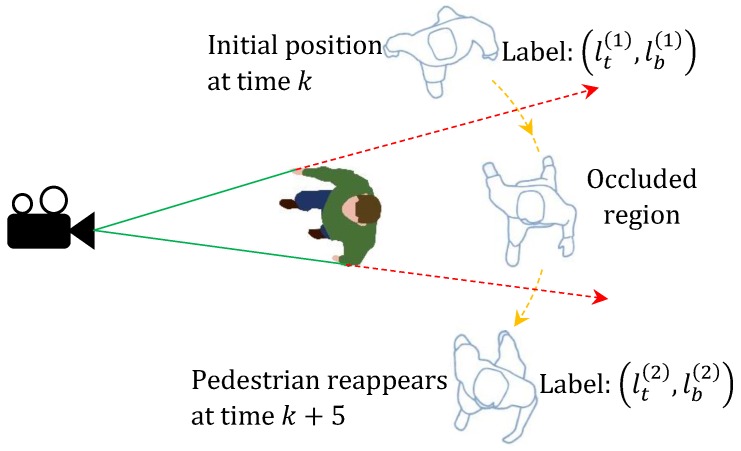
Person with initial label $\left(\ell_{t}^{\left(1\right)},\ell_{b}^{\left(1\right)}\right)$ is tracked until time *k*. The pedestrian is then occluded from time k+1 to time k+4, and re-tracked with label $\left(\ell_{t}^{\left(2\right)},\ell_{b}^{\left(2\right)}\right)$ from time k+5 onwards.

A scenario where occlusion leads to the creation of a new object label is depicted in Figure 1. The pedestrian with label $\left(\ell_{t}^{\left(1\right)},\ell_{b}^{\left(1\right)}\right)$ is tracked until it gets occluded by the other pedestrian at time *k*. When the pedestrian is in the occluded region, a corresponding measurement for that pedestrian will not appear in the set of measurements at times k+1 through k+4. During this period, the target detector will only return one measurement for both pedestrians. Due to the absence of a corresponding measurements for a significant number of time frames, the Bayesian tracking algorithm may discard the track of the occluded pedestrian. At time k+5, measurements for both pedestrians will appear in the set of point measurements. As such, the tracking algorithm will start to track the reappeared pedestrian, albeit with a new label $\left(\ell_{t}^{\left(2\right)},\ell_{b}^{\left(2\right)}\right)$. However, this is not acceptable in applications where identity of the pedestrians tracked are of high importance. We discuss this problem in a mathematical stand point below.

Consider an object with label $\ell_{m}$ that is accidentally missed (does not appear in the set of point measurements *Z*) at time *k*. The correct hypothesis (I,ξ,θ), should include $\ell_{m}$ in its hypothesised labels *I*, and its association map should be correct too, i.e., $\theta (\ell_{m})=0$.

For the ease of discussion, let us assume a constant probability of detection $p_{D}\left(x,\ell \right)=p_{D}$. From Equations (13) and (14), we simply deduce that $\eta_{Z}^{\left(\xi ,\theta \right)}\left(\ell_{m}\right)=1-p_{D}.$ Thus, based on Equation (11), through the update step, the weights of all the (correct) hypotheses that actually include the miss-detected object label would be reduced by a factor of $1-p_{D}$. A similar phenomenon will happen in the more general case where the detection probability varies with the object state and label. With an acceptably large detection probability, this can lead to a significant reduction of the weights of those hypotheses.

If an object is missed in multiple consecutive times, all the hypotheses that include the object will have significantly small weights, probably small enough for those to be pruned. This leads to permanent disappearance of the label of the object.

In radar tracking, specially in low Signal-to-Noise Ratio (SNR) scenarios, the targets can remain hidden from the from the radar for few consecutive frames [62]. In such cases, one can set the probability of detection to be relatively low so that those tracks remain within the filter over the course of target occlusion (or miss-detection) and then later be recovered. Furthermore, the method formulated in Inostroza et al. [63] can also be adopted to handle partial occlusions. However, in visual tracking, the occlusion period can be substantially longer as the objects are inherently not point-sized. Furthermore, in applications where visual tracking is normally employed, such as pedestrian tracking, two targets may walk together for long periods unlike in most of the radar tracking applications, such as tracking aircraft.

Depending on the frame rate of the imaging device, the occlusion period may take numerous frames during which, the object detection module may return only one measurement for the occluding objects. The GLMB filter in its standard form, will lose track of the target that is missed during the occlusion period, even after it separates from other targets and is re-detected (appears in the measurement set *Z*).

A remedy for the post-occlusion detections to be included in the filter outputs is to extend the birth process so that it includes all possible locations where missing objects may be re-detected. However, this can lead to the hypothesised newly born objects that are close to existing (and not-occluded, hence detected) objects being falsely accepted by the filter as existing (newly born) objects because they may match some detections. Such false alarms must be detected and removed from the filter outputs.

On the other hand, when an occluded then re-detected object is recovered, it will be given the label of a newly born object. Therefore, a label recovery mechanism is also needed to match the recovered object labels with one of the pre-occlusion objects. In the following sections, we present two intuitive solutions that handle the false alarm and label recovery issues within a GLMB filter used for multi-object tracking in video. The solutions are not only tailored for visual tracking scenarios (via effectively using colour and displacement information), but also are economic in terms of computational and memory requirements.

## 4. Proposed Method

To handle occlusions in multi-object visual tracking, we suggest a combination of false alarm detection and removal and label recovery algorithms that operate on the labeled set estimate returned by the GLMB filter at the conclusion of every filtering iteration. We note that in principle, the two operations would be formulated for implementation within the update step of the filter. However, such implementations would involve computation of a huge number of mutual distances and memorising a substantial number of colour histograms, thus too computationally expensive for online visual tracking. For example, assume that there are actually *m* number of targets at time *k*. However, in practice, there will be *n* number of tracks present in the track table with n>>m. Most of these *n* tracks will have low probabilities of existence and will get pruned due to the fact that they will most likely to be not supported by measurements in the next time steps. Note that in this instance, the "recent disappearance table" have to be updated to include these tracks. As such, we have to calculate and store a significantly large number of histograms for these tracks. While increasing the computational time of the algorithm, this may be erroneous as now we are most likely storing tracks which do not actually represent a true target.

**Figure 2:**
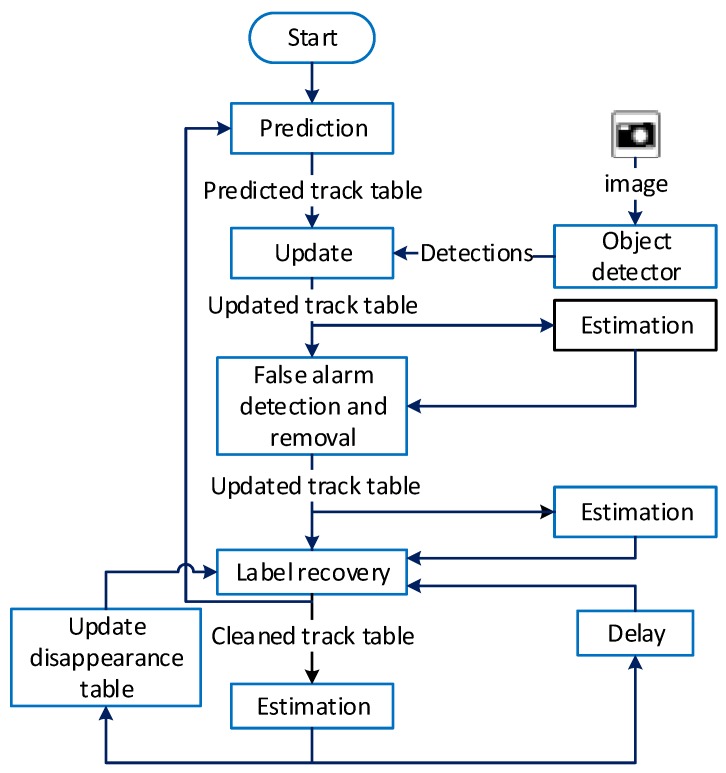
An overview of the proposed solution and its major components.

In contrast, our proposed method only needs to compute a limited number of mutual distances and memorise a few colour histograms, because it only operates on estimates and not the entire ensemble of labeled multi-object hypotheses in the GLMB posterior. An overview of the proposed solution and its major components are depicted in Figure 2. A detailed descriptions the proposed false alarm detection and removal, and label recovery methods are given below.

### 4.1. False Alarm Removal and Detection

Algorithm 1 shows a step-by-step pseudocode for our proposed approach to handle false alarms. Consider the multi-object estimate $\hat{X}$ returned by the filter-see Equation (18). The algorithm compares each object with all the other objects in the labeled set. For a labeled object $y^{\prime }=\left(x^{\left(\ell^{\prime }\right)},\ell^{\prime }\right)\in \hat{X}$ to be detected as false alarm and removed from the estimate, it should satisfy the following three conditions in terms of its similarities with another detected object $y=\left(x^{\left(\ell \right)},\ell \right)\in \hat{X}$:

1. The two objects must have substantial overlap.
2. y must be older than $y^{\prime }$.
3. The two objects must be similar in size (in terms of image pixels).

**Algorithm 1** Pseudocode for the proposed false alarm removal and detection algorithm.
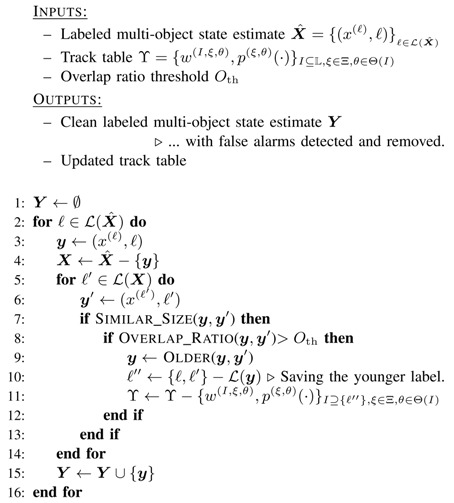


The rationale behind the first two conditions is that we are looking for false alarms that are caused by birth objects that match measurements, and existing objects match them well as well. Hence, each false alarm is expected to significantly overlap an existing object. Furthermore, being the result of expansion of birth process, the false alarms are expected to have been born after the real objects with which they have substantial overlap.

To implement the validation of the above conditions, we normally use comparison thresholds. For instance, “substantial overlap” is determined based on whether the overlapped area is greater than a given percentage of the larger object area and “similarity in size” is determined based on whether the width and height of the smaller object are within a given percentage of those of the larger object.

The algorithm searches for all pairs of objects in the estimate $\hat{X}$ that substantially overlap, and removes the one with newer label as a false alarm. There might be two real objects, one far from and the other close to the camera, and the closer object (larger in the image) may cover a substantial portion of the farther one. In this case, the algorithm should treat both objects are real, and no false alarm should be detected and removed. This is the main rationale behind the third condition.

If all the above three conditions are satisfied, the track for the object with the younger label will be removed. When the process arrives at the step 9 (of Algorithm 1), this means that there are two targets with similar size and substantial overlap. Thus, the target associated with the younger label from has to be removed from the track table $\Gamma_{k}={\left\{w_{k}^{\left(I,\xi ,\theta \right)},p_{k}^{\left(\xi ,\theta \right)\left(\cdot \right)}\right\}}_{I\subseteq I,\xi \in \Xi ,\theta \in \Theta (I)}$. This is achieved by:

- Step 9: Determine the target associated with the older label.
- Step 10: Use the above information to determine the label of the younger target and store it temporarily.
- Step 11: Remove the track associated with the younger target (as calculated in the two previous steps).

The functions Similar_Size(·,·), Overlap_Ratio(·,·), and Older(·,·) depend on the template used for representing the single objects and the construct of the single-object states in the filter. For instance, consider a scenario where each single-object is presented by a rectangular blob template and its unlabelled state is constructed as

$$x={\left[\begin{array}{cccccc} p_{x} & p_{y} & \dot{p_{x}} & \dot{p_{y}} & w & h \end{array}\right]}^{⊤},$$

where $p_{x}$ and $p_{y}$ denote the image coordinates of the centre of the blob (object location), and *w* and *h* denote the width and height of the blob, respectively.

The function Similar_Size(·,·) returns a true if the difference between the width and height of the two objects are each, less than a small portion (20% in our experiments) of the width and height of the smaller object. The function Overlap_Ratio(·,·) computes the overlapping area between the two rectangular blobs and returns its ratio to the smaller blob area. The threshold parameter $O_{\mathrm{th}}$ was chosen as 80% in our experiments.

Having the time of birth recorded as part of the object’s label, makes it straightforward to distinguish which of two objects is older. If $L\left(y\right)=\left(\ell_{t},\ell_{b}\right)$ and $L\left(y^{\prime }\right)=\left(\ell_{t}^{\prime },\ell_{b}^{\prime }\right)$, then we have:

(19) $$O\mathrm{LDER}(y,y^{\prime })=\left\{\begin{array}{cc} y & \mathrm{if}\;\ell_{t}<\ell_{t}^{\prime }\\ y^{\prime } & \mathrm{otherwise}. \end{array}\right.$$

After identifying the false alarms, the corresponding hypotheses should be removed from the track table of the filter. This guarantees that those false alarms will not be propagated to the next time step. This can be simply achieved with the help of the unique identities of the objects in the following manner. For each false alarm detected by Algorithm 1, its label is recorded and the matching track (hypothesis) in the track table of the filter is found and removed from the track table. The weights of the GLMB components are then re-normalised.

### 4.2. Label Recovery

As it was mentioned earlier, in many visual tracking applications, either due to the shortcomings of the employed detector or due to occlusion, object(s) may not be tracked and temporarily disappear from the trajectories returned by the GLMB filter. When an object is re-detected (e.g., after occlusion), the filter can include the object in its estimate but as a new trajectory (with an incorrect label). In some tracking applications such as surveillance, it is of paramount importance that the objects have consistent labels before and after such temporary disappearances. Inspired by the decay functions in distance dependent Chinese restaurant processes [64], we propose a novel label recovery module to consistently maintain the labels of the objects in occlusion and miss-detection events.

Our proposed label recovery solution is based on constructing a recent disappearance lookup table that holds all the objects that have disappeared during the past $k_{\max}$ times and have not reappeared yet. The parameter $k_{\max}$ is practically the maximum duration of occlusion that is expected to be handled by our method. The lookup table is constructed as follows.

Let us denote the multi-object estimate returned by the GLMB filter at time *k* by ${\hat{X}}_{k}$. For every single-object state $x\in {\hat{X}}_{k-1}$, it is considered as disappeared at time *k* if its label does not appear in the set of estimated labels at time *k*, i.e., $L\left(x\right)\notin L\left({\hat{X}}_{k}\right)$. In that case, the time of disappearance, *k*, the label of the object $L\left(x\right)=\left(\ell_{t},\ell_{b}\right)$, its location $\left(p_{x},p_{y}\right)$ and the colour histogram of the contents of the object represented by ***x*** in the image, denoted by *H*, are all stored in the lookup table. This means appending a new row to the bottom of the lookup table, with contents $\left[\begin{array}{cccccc} k & \ell_{t} & \ell_{b} & p_{x} & p_{y} & H \end{array}\right].$ To constrain its size, at any time *k*, all the recorded rows with birth time labels $\ell_{t}<k-k_{\max}$ are removed.

For label recovery, at any time *k*, we first find the set of all the newly born objects at time *k* among the estimates returned in ${\hat{X}}_{k}$,

(20) $${\hat{X}}_{B,k}=\{x\in {\hat{X}}_{k}\;|\;\;\exists \ell_{b}\in N;L\left(x\right)=\left(k,\ell_{b}\right)\}.$$

For each newly born object estimate ***x***, we then evaluate its similarity to each of the previously disappeared objects recorded in the recent disappearance lookup table. Let us assume that $\left(p_{x},p_{y}\right)$ is the location of ***x***, and *H* is its colour content histogram. Consider a previously disappeared object that is recorded in the *i*-th row of the lookup table as $\left[\begin{array}{cccccc} k_{i} & \ell_{i,t} & \ell_{i,b} & p_{x_{i}} & p_{y_{i}} & H_{i} \end{array}\right]$. We are interested in an intuitive and effective technique to quantify the likelihood of ***x*** representing the reappearance of the above recorded object. Hereafter, we denote this likelihood by $l_{i}\left(x\right)$.

In visual tracking applications, one would intuitively expect a disappearing object to maintain its visual appearance (hence its colour content histogram) when reappearing. The similarity in visual appearance can be quantified in terms of the distance between the two colour histograms. A common choice for formulating such a distance is the Bhattacharyya distance [10,65,66].

**Remark** **3.** *It should be noted that other image-based similarity measures, such as normalised correlation can be used in the algorithm. However, we used HSV colour histograms as they are shown to be robust for illumination variations [67]. While histograms are not robust to partial occlusion, they provide a simple and fast yet, substantially accurate image similarity measure. The adoption of other similarity measures in the proposed framework is relatively straightforward.*

In addition to similarities in colour contents, depending on the period of disappearance, there would be a constrained area in which the object can possibly reappear. Considering the most general model, the random walk, such an area is a disk around $\left(p_{x_{i}},p_{y_{i}}\right)$, with a diameter that is proportional to the hypothesised period of disappearance, $k-k_{i}$.

Based on the above constraints, we suggest to quantify the likelihood of ***x*** representing the *i*-th recorded disappearance in the lookup table, as follows:

(21) $$\begin{array}{ccc} l_{i}\left(x\right) & \propto & \beta \;\exp\left(-\frac{\sqrt{{\left(p_{x}-p_{x_{i}}\right)}^{2}+{\left(p_{y}-p_{y_{i}}\right)}^{2}}}{2{\left[\left(k-k_{i}\right)\sigma_{v}\right]}^{2}}\right)+\;\left(1-\beta \right)\;\exp\left(-\frac{d{\left(H,H_{i}\right)}^{2}}{2\sigma_{H}^{2}}\right), \end{array}$$

where $d(H,H^{\prime })$ denotes the Bhattacharyya distance between the two histograms, β∈[0,1] is the weight given to spatial component of the likelihood function, $\sigma_{v}$ is the scale of noise in random walk motion model in pixels, and $\sigma_{H}$ is the standard deviation of possible random changes in an object’s appearance (its colour content histogram) from one frame to another. Note that the weighted sum in the right hand side of Equation (21) is normalised.

The choice of β parameter depends on the application. For example, if there is no appearance information or all the objects of interest have the similar appearances, lower emphasis on the appearance component and more on the spatial component (larger β) is suitable. In cases where the objects can be easily distinguished from their colour features, one can assign a larger weight for the appearance component (smaller β).

The proposed label recovery algorithm appears in Algorithm 2. The first for loop detects the targets that disappeared in the previous time step, and amend the recent disappearance lookup table.
**Algorithm 2:** Pseudocode for the proposed label recovery algorithm.
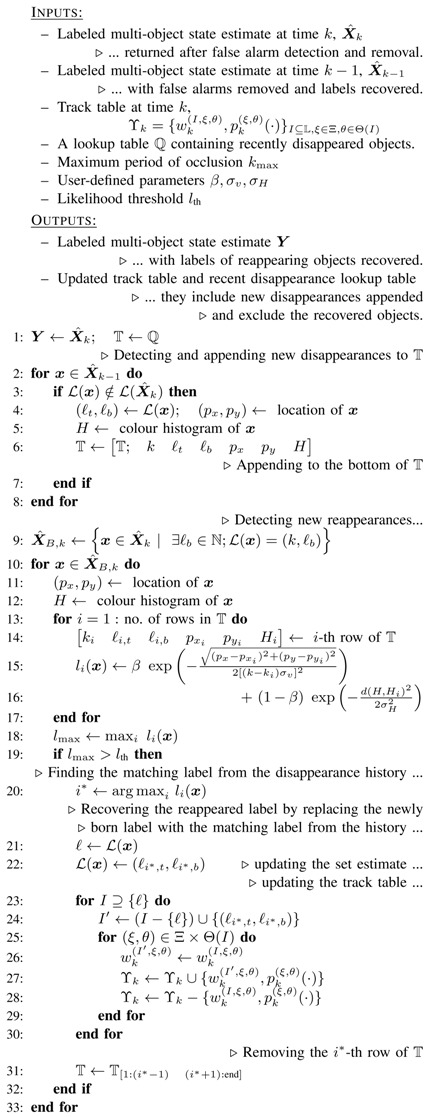


In the second for loop, for each element ***x*** in the newly born estimates, its likelihood to be a reappearance of all the previously disappeared objects is computed, and the best candidate (with the maximum likelihood) is chosen. If its likelihood is larger than a user-defined threshold $l_{\mathrm{th}}$, it is accepted as a reappearance, and its label is recovered. Note that the likelihood values in (21) are all normalised to fall within [0,1], the same is correct for the threshold $l_{\mathrm{th}}$, which was set to 0.70 in our experiments.

**Remark** **4.** *Both in Algorithm 1 and in Algorithm 2, in addition to the labels, the track table of the filter is updated. In Algorithm 1, once a false alarm is detected, its label is saved (see line 10) then all the hypotheses containing that label are removed from the track table (see line 11). Similarly, in Algorithm 2, when a reappearance is detected (by finding a newly born target being well matched with one of the recently disappeared targets–see lines 10–19), then not only the label of the corresponding estimate is updated (line 22), but also all the hypotheses in the track table which contain that newly born label are updated via substituting that label with the recovered label (lines 23–30).*

## 5. Experimental Results

### 5.1. Target Motion and Measurement Models

In extensive experiments using publicly available datasets, we examined the performance of our comprehensive visual tracking solution and compared it with the following state-of-the-art methods in the computer vision literature: RMOT [38], StruckMOT [68], GeodesicTracker [14], PRIMPT [69], Nonlinear motion [18], CemTracker [16], and KSP [70]. Our solution included the false alarm removal and label recovery algorithms integrated into the SMC implementation of the GLMB filter.

All the datasets included detection results, and for a fair comparison, we used the same detections used in the other methods. The detections were based on rectangular object templates. Hence, the unlabelled single-object state is formulated as $x={\left[\begin{array}{cccccc} p_{x} & p_{y} & {\dot{p}}_{x} & {\dot{p}}_{y} & w & h \end{array}\right]}^{⊤}$ where *w* and *h* are the width and height of the blob containing the object in the image.

Due to the perspective effect, the object sizes vary when they move towards or away from the camera. Thus, the width and height of the objects are set to have variable, but constrained values. The upper bound ensures that multiple objects are not represented by a single rectangular blob, and the lower bound is to make sure that the rectangular blob is large enough to represent a single object. The objects are set to have a constant survival probability of $p_{S_{k}}\left(\cdot \right)=0.99$.

All the case studies examined in our experiments involve pedestrian tracking. When walking, people tend to have a nearly constant velocity unless their motion is intermittently interfered due to various reasons such as suddenly stopping to talk to another person and going around an obstacle. Hence, we use a nearly constant velocity model for evolution of object state. The randomness in nearly constant model permits the hypothesised objects to change their velocities. This allows us to track objects when there are changes in the velocities and direction of movements.

In all case studies, the birth processes are labeled multi-Bernoulli with constant probabilities of existence of 0.03. In order to strike the right balance between accuracy of particle approximation and computation, the number of particles per object is constrained between $L_{\min}=100$ and $L_{\max}$ = 500.

### 5.2. Performance Metrics and Datasets

It is well known that different performance metrics can lead to different assessments for the same tracking results [71]. In order to permit a fair comparison we use the same set of metrics proposed by Li et al. [72], as those have been widely used in the visual tracking literature [18,19,38,69,73]. The tracking metrics are as follows:

– recall (REC - ↑): Correctly tracked objects over total ground truth;
– precision (PRE - ↑): Correctly tracked objects over total tracking results;
– false alarms per frame (FAF - ↓)
– percentage of objects tracked for more than 80% of their life time (MT - ↑);
– percentage of objects tracked for less than 20% of their life time (ML - ↓);
– percentage of partially tracked objects (PT ↓ = 1 - MT - ML);
– identity switches (IDS - ↓);
– the number of fragmentations (Frag - ↓) of ground truth trajectories.

Here, the symbol ↑ means that higher scores are better, and ↓ means that lower scores are preferred.

Further it should also be noted that most of the methods used in comparison have used the same detection results and ground truth available in the website (https://sites.google.com/site/boyanghome/downloads) of one of the authors of [18,19], along with the evaluation software.

We selected three publicly available datasets which are widely used in the literature [9,18,19,38,68,69,73,74] to benchmark the performance of visual tracking algorithms. These datasets were specifically selected to include video sequences recorded using both stationary and mobile cameras, to demonstrate that the proposed tracking method can be effectively applied in both scenarios. Furthermore, these sequences include periodically overlapping objects and thus the effectiveness of occlusion handling plays a key role in tracking performance.

The specific sequences used in experiments are

– view 1 of S2L1 sequence from PETS2009 dataset;
– TUD-Stadtmitte sequence from ETH dataset; and
– Bahnhof and Sunnyday sequences from ETH dataset.

The ability of occlusion recovery in our online algorithm is demonstrated using these sequences. Furthermore, automatic track management (i.e., initialisation, maintenance and deletion of object trajectories), tracking through clutter is also demonstrated.

It should be noted that although StructMOT [68] is labeled as an online method, a cost function should be trained offline which uses multiple features such as histogram of optical flow (HOF) and 2D motion information. Moreover PRIMPT [69] also needs to be trained for its appearance model. In contrast, our method only uses the detections and does not involve any training procedure. Furthermore, the nonlinear motion [18] and GeodesicTracker [14] methods are only formulated for stationary cameras, where as our method can be applied for both stationary and mobile cameras.

#### 5.2.1. PETS2009 S2L1 View1

This sequence is arguably the most widely used video sequence in evaluating visual tracking algorithms and it is recorded in an outdoor environment with a camera mounted in an elevated viewpoint. Tracking is required for objects with nonlinear motion and closely moving objects. Moreover, due to the perspective effect, object sizes can vary substantially. A light pole in the middle of the scene causes objects temporarily disappearing from detections. The birth process used for this sequence is composed of five labeled Bernoulli components. Since this sequence is recorded using a stationary camera, we assumed that the entrance points are known prior to applying the algorithm and thus we generated three labeled Bernoulli components at the three road entry points to the scene. The other two labeled Bernoulli components are generated uniformly throughout the image to re-detect the disappearing objects.

**Figure 3:**
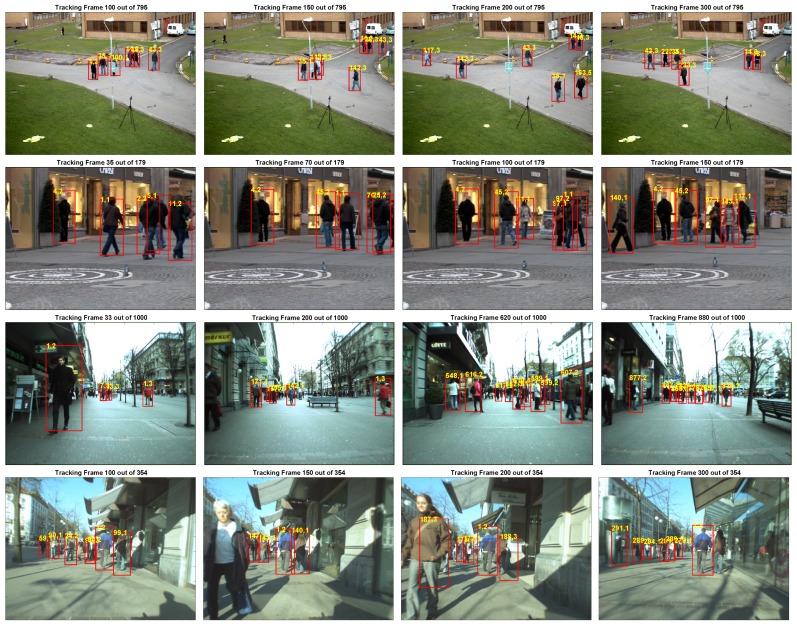
Tracking results for PETS2009S2L1V1, TUD-Stadtmitte, ETH Bahnhof and Sunnyday sequences. Examples for successful occlusion handling include pedestrians with labels; (142,2) in PETS2009S2L1V1—first row two middle images; (4,2) in TUD-Stadtmitte—second row all images; and (1,3) in ETH Bahnhof—third row first two images. Videos for the results presented are included as supplementary material.

From the comparative results presented in Table 2, it can be seen that our online method returns generally better values for precision, MT, ML, Frag and IDS metrics when compared to online methods. As previously mentioned, although StructMOT reports better results for REC and Frag metrics, it should be trained offline but our method only uses the detections. Figure 3 depicts snapshots of the tracking results for various video sequences.

#### 5.2.2. TUD-Stadtmitte

This outdoor video sequence is recorded in a busy street with a very low view point and with many occluded pedestrians. While this sequence is also recorded with a stationary camera, we modelled the birth process by considering four labeled Bernoulli components with their locations uniformly distributed throughout the image. The rationale behind this birth process is that it will permit the tracking in presence of a large number of occlusions due to the low camera view point.

Most of the pedestrians have almost similar colour features resulting in similar colour histograms. Thus motion information is more important than colour information in tracking. We assigned a large value for the weight of motion information, β parameter, in our occlusion label recovery algorithm. Here, all the methods report a lower recall value and higher partially tracked value due to the fact that one of the objects remain occluded for almost its entire life in the video and two objects that appear towards the end of the sequence are not included in the ground truth reported in the dataset.

It is evident that on this dataset, the performance of our method is better than or similar to the state of the art methods in all the metrics. Particularly, there has been no ID switches which demonstrate excellent label management performance. In addition, the detections for this sequence occasionally include multiple detections for the same objects. The ability to handle clutter in GLMB filter mitigates the affects of these multiple detections.

#### 5.2.3. ETH Bahnhof and Sunnyday

ETH dataset is obtained by a camera mounted on a mobile platform with a very low viewpoint. Both sequences used in this study consist of a number of occluded pedestrians and detector failures as well. The birth process in both sequences is modelled by four labeled Bernoulli components with their locations distributed throughout the image. Since the birth model is multi-Bernoulli, having four birth components will allow us to detect up to four new objects at each frame.

From the results reported in Table 2, it can be seen that our method is performing better than or comparable to the state-of-the-art methods in all metrics. The metric values for these sequences are lower compared to that of the other two sequences as there is a large number of occluded objects and miss-detections (specially in the Sunnyday sequence). In both sequences, when the reflection of a pedestrian appears on the glass, it is detected by the detector and thus tracked by our method, resulting in lower metric values. Furthermore, frequent miss-detections make the fragmentation metric higher.

### 5.3. Ablation Study

A comprehensive ablation study was performed to establish the performance of the two proposed modules. We use the standard GLMB filter as the baseline algorithm and performed the experiments on the PETS2009 S2L1V1 and TUD-Stadtmitte sequences. The results of the ablation study is presented in Table 3. The two proposed modules are abbreviated as FADR and LR, which stand for False Alarm Detection and Removal, and Label Recovery. The results show that the proposed false alarm detection and removal algorithm has reduced the FAF (False Alarms per Frame) metric significantly. This shows the effectiveness of the proposed module in handling false alarms. It can be also observed that the addition of label recovery algorithm has substantially reduced the fragmentation and ID switching errors.

### 5.4. Computational Cost

Comparing the computation cost of different algorithms is difficult as software implementation and hardware configuration play a significant role in such comparisons. The proposed algorithm is implemented in MATLAB R2015a running on a core i7 laptop with 8 GB of memory without focusing on speed optimisation. With the particle count mentioned in Section 5 and LMB birth processes mentioned in separate sections for each dataset, the algorithm is capable of achieving a speed of 12 frames per second (fps) for sequences with comparatively low number of targets such as PETS2009-S2L1V1. It can also achieve 4 fps run time for the datasets with comparatively high number of objects such as ETH Sunyday sequence. Such computational costs permit the proposed algorithm to be used in real-time applications. The competing online methods, such as StrcukMOT, RMOT and Geodesictracker are reported to have 15 fps, 37 fps and 11.2 fps speeds respectively for sequences with low number of objects on systems with different software and hardware configurations.

## 6. Conclusions and Future Work

A novel method for multi-target tracking in video was proposed. The method is designed based on the GLMB filter with modifications to the track table of the filter integrated in such a way that the number of targets and their states and labels can be estimated and propagated in each frame in real time. The resulting method enables the GLMB filtering core of the tracker to deal with targets that are of finite and time-varying sizes, and can occlude each other intermittently during the tracking period. One key contribution is to extend the birth process model so that it covers all the regions within the state space where targets can occlude each other. This leads to the filter being capable of re-detecting the targets which have disappeared during an occlusion period and including them within its tracks, after occlusion. The above mentioned extension of the birth process leads to false alarms. The second key contribution is an intuitive method to detect and remove such false alarms, and update the filter’s track table accordingly. The third significant contribution is an intuitive algorithm to recover the label of an occluded target after it reappears, via introduction of a recent disappearance track history. Step-by-step pseudocodes of the proposed algorithms were presented in detail. Comparative experiments involving several challenging (and commonly used for benchmarking) visual tracking datasets demonstrated that our method outperforms, or performs similar to, the state-of-the-art in terms of various common tracking metrics.

As a future work, utilising different similarity measures such as normalised cross correlation [75] for both false alarm detection and label recovery algorithms can be investigated. When selecting such a similarity measure, factors such as computational cost, robustness to partial occlusions and robustness to illumination variations have to be considered. However, to detect false alarms we must still use the size and label-related information associated with the targets to ensure that objects with similar appearances are also distinguished and tracked. Furthermore, the label recovery procedure can be extended to be utilised in a multi-camera setting with non-overlapping fields of view, given that the camera intrinsic and extrinsic parameters are provided. The detections in image coordinates need to be projected into 2D or 3D real world coordinates due to the fact that the proposed label recovery algorithm relies on constraining the area of a possible reappearance of an object after its disappearance. This cannot be achieved in the image coordinate domain in a multi-camera setting due to the difference in the fields of view of the cameras.

## Figures and Tables

**Table 1:** Notations and abbreviations used in this paper.

Notation/Abbreviation	Description
B	Label space of new born targets
C	Index space
L	Label space of existing objects
N	Space of natural numbers
R	Space of real numbers
X	State space
Z	Measurement Space
*L*	Set of track labels
*X*	Set of state vectors
**X**	Set of labeled state vectors
*Z*	Set of measurements at
	time k+1
$Z_{1:k+1}$	Set of all measurements
	up to time k+1
RFS	Random Finite Sets
LMB	Labeled Multi-Bernoulli
GLMB	Generalized labeled
	Multi-Bernoulli
SMC	Sequential Monte Carlo

**Table 2:** Comparative results for PETS2009-S2L1V1, TUD-Stadtmitte and, ETH Bahnhof and Sunnyday.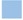 represents online methods.

Dataset	Method	REC ↑	PRE ↑	FAF ↓	GT	MT ↑	PT ↓	ML ↓	Frag ↓	IDS ↓
PETS09-S2L1	GLMB (Ours)	93.3%	96.9%	0.17	19	94.7%	5.3%	0.0%	20	0
	StruckMOT [68] (*o.t.*)	97.2%	93.7%	0.38	19	94.7%	5.3%	0.0%	19	4
	RMOT [38]	96.9%	97.4%	0.15	19	89.5%	10.5%	0.0%	7	2
	GeodesicTracker [14]	-	-	-	23	100.0%	0.0%	0.0%	16	9
	Nonlinear motion [18] (*s.c.*)	91.8%	99.0%	0.05	19	89.5%	10.5%	0.0%	9	0
	CemTracker [16]	-	-	-	19	94.7%	5.3%	0.0%	15	22
	KSP [70]	-	-	-	23	73.9%	5.3%	0.1%	22	13
TUD - Stadtmitte	GLMB (Ours)	87.1%	97.1%	0.16	10	80.0%	20.0%	0.0%	6	0
	StruckMOT [68] (*o.t.*)	87.3%	95.4%	0.25	10	80.0%	20.0%	0.0%	11	0
	RMOT [38]	87.9%	96.6%	0.19	10	80.0%	20.0%	0.0%	7	6
	PRIMPT [69] (*o.t.*)	81.0%	99.5%	0.028	10	60.0%	30.0%	10.0%	0	1
	OnlineCRF [19]	87.0%	96.7%	0.18	10	70.0%	30.0%	0.0%	1	0
	CemTracker [16]	-	-	-	10	40.0%	60.0%	0.0%	13	15
	KSP [70]	-	-	-	9	11.0%	5.3%	0.1%	15	5
ETH Bahnhof and Sunnyday	GLMB (Ours)	77.1%	83.6%	1.161	124	54.0%	40.3%	5.6%	91	31
	StruckMOT [68] (*o.t.*)	78.4%	84.1%	0.98	124	62.7%	29.6%	7.7%	72	5
	RMOT [38]	81.5%	86.3%	0.98	124	67.7%	27.4%	4.8%	38	40
	MT-TB [73]	78.7%	85.5%	-	125	62.4%	29.6%	8.0%	69	45
	PRIMPT [69] (*o.t.*)	76.8%	86.6%	0.89	125	58.4%	33.6%	8.0%	23	11
	OnlineCRF [19]	79.0%	85.0%	0.64	125	68.0%	24.8%	7.2%	19	11
	CemTracker [16]	77.3%	87.2%	-	124	66.4%	25.4%	8.2%	69	57

Note: The arrow symbol ↑ represents that higher scores indicate better results and ↓ represents that lower scores indicate better tracking results.

**Table 3:** Ablation study. Results for baseline (GLMB), GLMB + FADR and GLMB + FADR + LR algorithm on PETS2009 S2L1V1 and TUD-Stadtmitte sequences.

Dataset	Algorithm	REC ↑	PRE ↑	FAF ↓	GT	MT ↑	PT ↓	ML ↓	Frag ↓	IDS ↓
PETS2009-S2L1V1	GLMB	92.1%	90.4%	0.30	19	94.7%	5.3%	0.0%	44	9
	GLMB + FADR	92.4%	96.0%	0.17	19	94.7%	5.3%	0.0%	42	10
	GLMB + FADR + LR	93.3%	96.9%	0.17	19	94.7%	5.3%	0.0%	20	0
TUD-Stadtmitte	GLMB	84.9%	90.1%	0.35	10	80.0%	20.0%	0.0%	12	14
	GLMB + FADR	85.8%	97.0%	0.17	10	80.0%	20.0%	0.0%	13	13
	GLMB + FADR + LR	87.1%	97.1%	0.16	10	80.0%	20.0%	0.0%	6	0

## Supplementary Materials

The following are available online at https://www.mdpi.com/1424-8220/20/3/929/s1.
