# Supplementary material for: On-Line Visual Tracking with Occlusion Handling
Source: Sensors (Basel). 2020 Feb 10;20(3):929. doi: 10.3390/s20030929 (PMC7039229; doi:10.3390/s20030929)
Supplement: Supplementary file 1 [file sensors-20-00929-s001.zip › Supplementary Materials/Video Sequences - Description.pdf]

The provided supplementary files show the results of our algorithm applied to PETS2009-S2L1, TUDS-Stadtmitte, ETH Bahnhof and Sunnyday sequences.
Following table lists example instances where our algorithm was able to recover the labels of the missed or occluded objects include:

Dataset	Assigned label	Last correct estimate	Start	End	Label recovered at
PETS2009-S2L1	117,3 (M)	149	150	156	157
PETS2009-S2L1	1,3 (M)	107	108	110	111
PETS2009-S2L1	425,3 (M)	73	474	484	385
ETH Sunnyday	1,2 (M)	135	136	143	144
ETH Bahnhof	548,1 (ML)	611	612	617	618
ETH Bahnhof	605,3 (MR)	729	730	738	739

(M), (ML) and (MR) refer to different areas of the image frame. These can be used to locate the target.
(M) - Both horizontally and vertically middle portion of the frame
(ML) - Vertically middle and horizontally left portion of the frame
(MR) - Vertically middle and horizontally right portion of the frame

Please note that this is not a comprehensive list and does not included all the instances where our algorithm was able to recover the labels of the miss-detected or occluded targets.

Also note that the ability of our algorithm to detect and remove false alarms cannot be demonstrated through these video sequences. Reader is directed to refer table 3 of the manuscript to observe how our proposed algorithm has handled the false alarms (in terms of FAF metric).
